# Generation and characterization of a tetraspanin CD151/integrin α6β1-binding domain competitively binding monoclonal antibody for inhibition of tumor progression in HCC

**DOI:** 10.18632/oncotarget.6833

**Published:** 2016-01-07

**Authors:** Ai-Wu Ke, Peng-Fei Zhang, Ying-Hao Shen, Ping-Ting Gao, Zhao-Ru Dong, Chi Zhang, Jia-Bin Cai, Xiao-Yong Huang, Chao Wu, Lu Zhang, Qiang Kang, Li-Xin Liu, Nan Xie, Zao-Zhuo Shen, Mei-Yu Hu, Ya Cao, Shuang-Jian Qiu, Hui-Chuan Sun, Jian Zhou, Jia Fan, Guo-Ming Shi

**Affiliations:** ^1^ Liver Cancer Institute, Zhongshan Hospital, Fudan University, Key Laboratory of Carcinogenesis and Cancer Invasion (Fudan University), Ministry of Education, Shanghai 200032, P.R. China; ^2^ Department of Hepatobiliary Surgery, Subei People's Hospital, Yangzhou University, Yangzhou 225000, China; ^3^ Department of Hepatobiliary Surgery, Second Affiliated Hospital of Kunming Medical University, Kunming 650101, China; ^4^ Cancer Research Institute, Xiangya School of Medicine, Central South University, Hunan 410008, China

**Keywords:** hepatocellular carcinoma, tetraspanin CD151, integrin α6β1, monoclonal antibody, therapeutical agent

## Abstract

Our previous studies revealed that tetraspanin CD151 plays multiple roles in the progression of hepatocellular carcinoma (HCC) by forming a functional complex with integrin α6β1. Herein, we generated a monoclonal antibody (mAb) that dissociates the CD151/integrin α6β1 complex, and we evaluated its bioactivity in HCCs. A murine mAb, tetraspanin CD151 (IgG1, called CD151 mAb 9B), was successfully generated against the CD151-integrin α6β1 binding site of CD151 extracellular domains. Co-immunoprecipitation using CD151 mAb 9B followed by Western blotting detected a 28 kDa protein. Both immunofluorescent and immunohistochemical staining showed a good reactivity of CD151 mAb 9B in the plasma membrane and cytoplasm of HCC cells, as well as in liver cells. *In vitro* assays demonstrated that CD151 mAb 9B could inhibit neoangiogenesis and both the mobility and the invasiveness of HCC cells. An *in vivo* assay showed that CD151 mAb 9B inhibited tumor growth potential and HCC cells metastasis. We successfully produced a CD151 mAb 9B targeting the CD151/integrin α6β1-binding domain, which not only can displayed good reactivity to the CD151 antigen but also prevented tumor progression in HCC.

## INTRODUCTION

Hepatocellular carcinoma (HCC) is the fifth most common malignant neoplasm worldwide [1]. Disease outcomes have been attributed to (1) early distant metastasis and high rates of recurrence after intervention [2] and (2) a lack of effective and curative interventions [3, 4]. An increased understanding of the molecular mechanisms underlying this disease may give us insight into potential targets for developing new preventive and therapeutic options for HCC [5].

Tetraspanin CD151, one of the most important members of the tetraspanins, has been identified as an important player in physiological processes [6] and the progression of malignant tumors [7], including skin [8], pancreas [9], lung [10], colon [11] and breast tumors [12, 13]. Consistent with relevant reports, our serial studies implicated CD151 in several pathological processes, including tumor cell mobility [14], tumor neo-angiogenesis [15] and epithelial-mesenchymal transition (EMT) [4] in HCCs. A distinct feature of CD151 is its ability to self-assemble or associate with other transmembrane molecules to form tetraspanin-enriched microdomains (TEM) [6]. Among the associated partners, integrins are the principal group of proteins that interact with tetraspanin CD151 [6]. Previously, we also identified a group of partners that associated with CD151 in the HCC cell line HCCLM3 [16]. Additionally, we validated the role of the CD151/integrin α6β1 complex in the progression of HCC [16]. Therefore, both CD151 and CD151-enriched microdomains appears to be promising targets in the treatment of HCC [17]. However, simple inhibition of CD151 in HCC is evidently inappropriate because CD151 plays essential roles in normal physiological processes, including cell adhesion, motility, activation and proliferation [6, 18–20]. Based on the above evidence, the dissociation of CD151-depedent TEM could be an effective strategy for inhibiting CD151's tumor-promoting abilities without disrupting its physiological functions [17].

Molecular targeted therapies have provided researchers with a broader perspective on the management of cancer, including colorectal and non-small cell lung cancers [21, 22]. Clinical trials have demonstrated that sorafenib, a multikinase inhibitor against Raf-1, B-Raf, VEGFR2, PDGFR and c-Kit receptors, improves progression-free survival in advanced HCC [23]. The benefit derived from sorafenib for advanced HCC patients is significant, but the drug prolongs mean survival by only 3 months compared with placebo [23]. Moreover, adverse events such as diarrhea, fatigue, weight loss, and hand-food skin reactions are frequent and limit the use of sorafenib for HCC patients. Therefore, it is important to develop alternative monoclonal antibodies for HCC [24]. In this study, we generated a CD151 mAb 9B (IgG1, κ) against the CD151/integrin α6β1-binding domain and determined its bioactivity in HCC cells.

## RESULTS

### Characterization of CD151 mAb 9B

**Figure 1:**
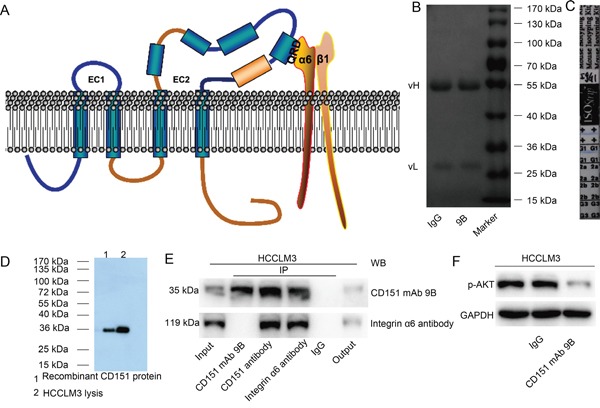
Characterization of CD151 mAb 9B **A.** Schematic representation of the anti-human CD151 mAb 9B against the CD151/integrin α6β1-binding site. **B.** SDS-PAGE and Coomassie brilliant blue for the anti-human CD151 mAb 9B. **C.** The immunotype of CD151 mAb 9B. **D.** Western blot analysis for CD151 in HCCLM3 cells and recombinant CD151 protein using CD151 mAb 9B. **E.** Co-IP assay using anti-human CD151 mAb 9B in HCCLM3 cells. **F.** CD151 mAb 9B inhibits Akt signaling in HCCLM3 cells.

A mouse anti-human CD151 mAb against the CD151/integrin α6β1 binding site [25] (Figure 1A, QRD^194-196^ site) was successfully produced and designated as CD151 mAb 9B. SDS-PAGE and Coomassie brilliant blue revealed two bands, including a light chain at 28 kDa and a heavy chain at 52 kDa (Figure 1B). The immunoisotype of CD151 mAb 9B was identified as IgG1 (Figure 1C). Western blotting using CD151 mAb 9B as the first antibody showed a band at 28 kDa in both HCCLM3 cell lysates and recombinant CD151 proteins (Figure 1D). A co-immunoprecipitation (IP) assay also showed that CD151 mAb 9B effectively immunoprecipitated CD151 protein as efficiently as the anti-CD151 antibody purchased from Abcam, which recognizes a different epitope of CD151. Interestingly, the integrin α6 protein cannot be detected by Western blotting from immuno-complex mixtures immunoprecipitated by CD151 mAb 9B, whereas integrin α6 can be detected from immuno-complex mixtures immunoprecipitated by the other anti-CD151 antibody. These results may indicate that CD151 mAb 9B competitively binds to the epitope through which integrin α6 binds to CD151 (Figure 1E). It was confirmed in our previous study that CD151 forms a complex with integrin α6, inducing Akt signaling. The results of the current study showed that treatment with 0.2 mg/ml of CD151 mAb 9B significantly decreased p-Akt expression in HCCLM3 cells (Figure 1F).

### Localization and expression of CD151 protein in HCC cells and tissues

**Figure 2:**
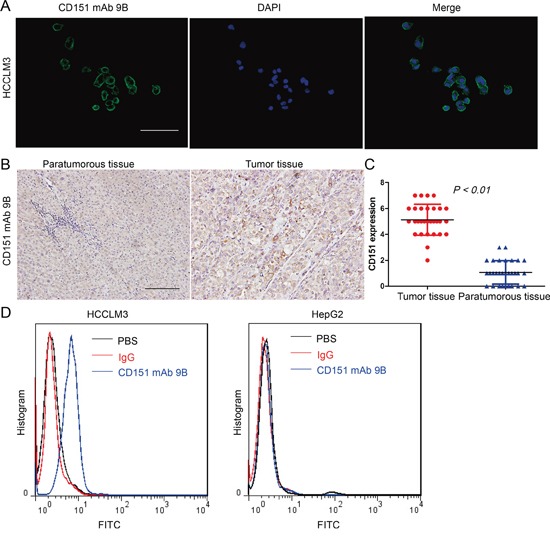
Localization and Expression of CD151 Protein in HCCs **A.** Immunofluorescent staining with CD151 mAb 9B in HCCLM3 cells. **B.** Immunohistochemical staining with CD151 mAb 9B in HCC tissues. **C.** Statistical analysis of CD151 expression in HCC tissues. **D.** FACS analysis of CD151 mAb 9B binding to the QRD^194-196^ conformational epitope of CD151 in HCC cells.

Immunofluorescent staining of HCCLM3 cells using the CD151 mAb 9B revealed that CD151 is expressed in the plasma membrane of tumor cells (Figure 2A). CD151 protein expression was detected in 30 pairs of HCC tumor and nontumor samples using immunohistochemical staining with CD151 mAb 9B. The results also showed that the immunoreactivity of CD151 was localized to the plasma membrane of the tumor cells and that the intensity of CD151 immunoreactivity in the HCC tissues was stronger than that in the paratumoral samples (Figure 2B and C, *p*<0.01).

A FACS assay was performed to test the binding efficiency of CD151 mAb 9B with the conformational epitope of CD151 QRD^194-196^ in HCC cells. The results indicated that CD151 mAb 9B bound to CD151 in the HCCLM3 cells efficiently; however CD151 mAb 9B did not bind to any proteins in the extracts from the HepG2 cells, which express low levels of CD151 (Figure 2D).

### CD151 mAb 9B Inhibited mobility, invasiveness and vascular remodeling in HCCs

**Figure 3:**
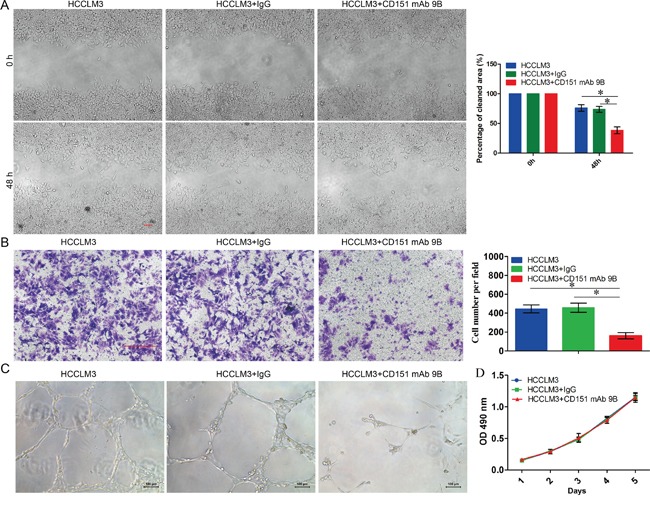
The effect of CD151 mAb 9B on HCCs **A.** Wound healing assay for the effect of CD151 mAb 9B on the mobility of tumor cells. **B.** Transwell assay for the effect of CD151 mAb 9B on the invasiveness of tumor cells. **C.** Matrigel angiogenesis to assay the effect of CD151 mAb 9B on the invasiveness of tumor cells. **D.** MTT assay for the effect of CD151 mAb 9B on the invasiveness of tumor cells. *P < 0.05.

To determine the bioactivity of CD151 mAb 9B, we performed an *in vitro* cell migration assay to assess its role in the mobility of tumor cells. The result showed an apparent decrease in the migratory ability of HCC cells treated with 0.2 mg/ml of CD151 mAb 9B. Representative photography indicated accelerated wound closure in the control cells (Figure 3A). This indicated that the migratory ability of the HCC cells was markedly suppressed after the administration of CD151 mAb 9B. Next, a transwell assay was used to investigate the role of CD151 mAb 9B in the invasiveness of tumor cells. The result showed that the average number of invaded cells significantly decreased after treatment with 0.2 mg/ml of CD151 mAb 9B compared with that of the control cells (Figure 3B, *p*<0.05). Third, a matrigel angiogenesis assay demonstrated that significantly more integrated capillary-like structures were found in the HUVECs cultured with DMEM and 10% fetal bovine serum than in the HUVECs supplemented with 0.2 mg/ml of CD151 mAb 9B (Figure 3C). However, we further determined the effect of the CD151 mAb 9B on the growth of the HCC cells and found that CD151 mAb 9B does not affect the proliferation of HCC cells (Figure 3D, *p*>0.05).

### CD151 mAb 9B inhibited neoangiogenesis and tumor growth and attenuated lung metastasis of HCC cells

**Figure 4:**
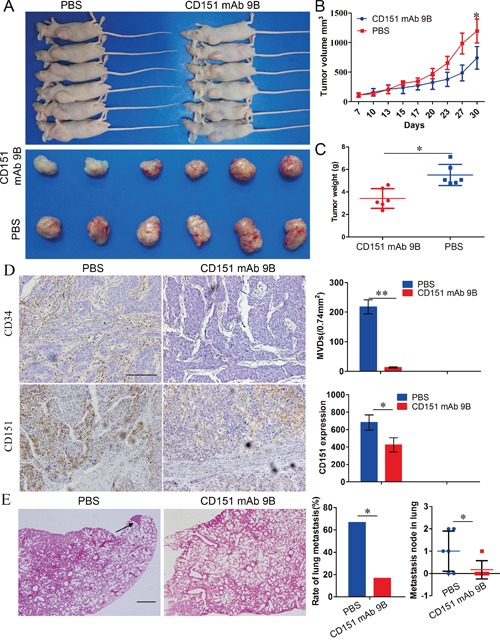
CD151 mAb 9B Inhibited the progression of HCCs *in vivo* **A, B** and **C.** Subcutaneous metastasis model to assay the effect of CD151 mAb 9B on the growth of tumor cells. **D.** IHC staining for CD34 and CD151 to assay the effect of CD151 mAb 9B on neoangiogenesis. **E.** Lung metastasis model to assay the effect of CD151 mAb 9B on the metastasis. *P< 0.05, **P< 0.01.

To further investigate the role of CD151 mAb 9B *in vivo*, we used a subcutaneous metastasis model to assay its impact on the efficiency of tumor growth and progression. The results revealed that the tumor volume and weight of the HCCLM3 cell-derived xenografts treated with 25 mg/kg CD151 mAb 9B was much lower than that in of the HCCLM3 cell-derived xenografts treated with PBS (Figure 4A, B and C;741.3 ± 191.4 mm^3^ *vs.* 1195.2 ± 202.5 mm^3^, 3.42 ± 0.88 g *vs.* 5.51 ± 0.93 g).

The microvessel density (MVDs) of HCCLM3 cell-derived xenografts treated with PBS were 217.69 ± 24.13/0.74 mm^2^ and were larger than those of the HCCLM3 cell-derived xenografts treated with 25 mg/kg CD151 mAb 9B (12.68 ± 2.47/0.74 mm^2^) (Figure 4D, *p*<0.01). CD151 expression in the HCCLM3 cell-derived xenografts treated with PBS was stronger than in the HCCLM3 cell-derived xenografts treated with 25 mg/kg CD151 mAb 9B (Figure 4D, *p*<0.05). These results indicate that CD151 mAb 9B inhibits neoangiogenesis through blockage of CD151-dependent TEM in HCC tissues.

Pulmonary metastases occurred in 66.7% (4/6) of the PBS group. However, a lower rate was observed in the CD151 mAb 9B group (16.7%, 1/6; Figure 4E). These data indicate that CD151 mAb 9B can inhibit tumor growth and the progression of HCC cells *in vivo*.

## DISCUSSION

Previously, our studies showed that CD151 is an important player in several processes during the progression of HCC [4, 14–16] and is considered an ideal therapeutic target for HCC [4, 14–17]. In the present study, we successfully produced a CD151 mAb 9B targeting tetraspanin CD151.

Intriguingly, the expression of CD151 was significantly reduced after CD151 mAb 9B treatment *in vivo*, which would be expected to result in reductions of both p-Akt and CD34 expression, as well as a reduced rate of lung metastasis. The alteration of these downstream molecular signals and phenotypes was similar to that observed in CD151 knockdown HCC cells [26]. It should be noted that a similar assay was applied *in vitro* as well. The above similarity suggests that CD151 mAb 9B exerts its antitumor effect though competitive binding to the tetraspanin CD151/integrin α6β1-binding domain.

In a previous study, we identified a set of proteins associated with CD151 in HCCLM3 cells (Established in Liver Cancer Institute, Zhongshan Hospital) and identified an important role for the CD151/integrin α6β1 complex in the progression of HCC [16]. Therefore, CD151-dependent TEM appears to be promising therapeutic targets for HCC [17]. Given that CD151 implicates in physiological processes, such as cell adhesion, motility, activation and proliferation [6, 18–20], simple blockage of CD151 in HCC is evidently inappropriate. Based on the above evidence, the dissociation of CD151-depedent TEM could be an effective strategy for inhibiting CD151's tumor-promoting abilities without disrupting its physiological functions [17]. Recent studies have shown that the QRD^194–196^ site of CD151 was required for binding with integrin α6β1 and its epitope [25]. In the present study, we chemically synthesized peptides of the CD151/integrin α6β1-binding domain (GQRDHASNIYKVEGGC) and then successfully produced a CD151 mAb 9B with a molecular weight of 28kDa. Second, CD151 mAb 9B displayed good reactivity to the CD151 antigen in HCCs. The newly synthesized antibody not only accurately reflected the intensity of the CD151 antigen by Western blotting but also correctly displayed the localization of the CD151 antigen by immunofluorescent and immunohistochemical staining, which indicates that it can be used in detection of the expression and localization of CD151 antigen in basic research. Third, CD151 mAb 9B showed good bioactivity for HCCs. On one hand, the newly synthesized antibody significantly inhibited the mobility and invasiveness of HCC cells *in vitro*. On the other hand, CD151 mAb 9B also inhibited growth and lung metastasis *in vivo*. Interestingly, CD151 mAb 9B had a minimal effect on the proliferation of HCC cells *in vitro*. These data indicate that CD151 mAb 9B plays an important role in the progression of HCC by targeting the CD151/integrin α6β1-binding; therefore, CD151 mAb 9B could overcome the limitation of CD151 blockage in HCC.

In conclusion, we successfully produced a CD151 mAb 9B that competitively binds to the tetraspanin CD151/integrin α6β1-binding domain. This mAb may be widely used for the detection of the expression and localization of the CD151 antigen in HCCs. More importantly, CD151 mAb 9B may be an efficient method for inhibiting of the progression of HCCs.

## MATERIALS AND METHODS

### Cell lines and animals

HCC cell lines HCCLM3, MHCC97-L (established in Liver Cancer Institute, Zhongshan Hospital [27]), HepG2 (American Type Culture Collection) and SP2/0 myeloma cells (American Type Culture Collection) were used in this study and routinely raised. Male BALB/c mice (8 weeks old) and male athymic BALB/c nude mice (4 weeks old) were purchased from the Shanghai Institute of Material Medicine and raised in specific pathogen-free conditions. Animal care was provided according to the guidelines established by the Zhongshan Hospital Experimental Animal Care Commission.

### Sample collection

Thirty pairs of fresh tumor and corresponding nontumor samples were collected from consecutive HCC patients who underwent curative resection at Zhongshan Hospital. All HCC samples were identified by histopathological examination. Of these 30 patients, 26 patients had a history of hepatitis B. No patients received preoperative anticancer treatment. Ethical approval was provided by the research ethics committee of Zhongshan Hospital, and written informed consent was obtained from each patient.

### Preparation and purification of anti-CD151 monoclonal antibodies

The monoclonal antibody was produced as previously described [28]. Briefly, small peptides were designed according to the CD151/integrin α6β1-binding domain (GQRDHASNIYKVEGGC), chemically synthesized and coupled with keyhole limpet hemocyanin (KLH). BALB/c mice were immunized with CD151/integrin α6β1 binding domain recombinant peptides. Splenocyte fusion was performed with SP2/0 myeloma cells, from which hybridomas of the desired characteristics were subsequently selected. The antibody was purified by protein G. The immunoglobulin subtype of the antibody was determined using an antibody isotyping kit (Roche Molecular Biochemicals, Indianapolis, IN, USA). The purified mAb was named CD151 mAb 9B and used for subsequent experiments.

### Western blotting, co-immunoprecipitation (co-IP), immunofluorescent (IF) and immunohistochemical (IHC) staining

Western blotting was performed as previously described [15]. CD151 mAb 9B (1:2000) was used to detect the expression of CD151 in three HCC cell lines and tumor samples from 30 patients. β-actin (1:5,000; Chemicon, USA) was used as an internal control. Co-IP was conducted as previously described [16]. Five milligrams of CD151 mAb 9B was used to extract CD151 from the HCCLM3 lysates (250 mg). The gel was stained using the PlusOne Coomassie brilliant blue (Amersham Biosciences). Immunofluorescent staining was performed as previously described [14]. The expression and localization of CD151 protein in HCCLM3 cells were assayed using the CD151 mAb 9B (1:200). The immunohistochemistry protocols are described elsewhere [14]. CD151 mAb 9B (1:200) detected the expression of CD151 in the HCC samples. Positive staining was measured as described in our previous report [14].

### FACS analysis

A total of 1 × 10^6^ cells were collected by centrifugation and incubated with 20 μg/ml of either CD151 mAb 9B or IgG in PBS containing 1% newborn calf serum for 45 min at 4°C. After being washed with cold PBS three times, the cells were incubated for an additional 45 min at 4°C with a FITC-conjugated secondary antibody (Kang-Chen Bio-tech, Shanghai, China) in the dark. The cells were then analyzed by FACS cytometry (Beckman Coulter Epics Altra, Miami, FL).

### Wound healing, invasion assay and matrigel angiogenesis assay

A wound-healing assay was used to evaluate the effect of CD151 mAb 9B on cell migration [29]. These cells were raised in serum-free medium supplemented with or without 0.2 mg/ml CD151 mAb 9B. The invasion assay was performed as described elsewhere [14]. The supernatant of NIH3T3 in DMEM supplemented with 10% fetal bovine serum with or without 0.2 mg/ml CD151 mAb 9B, which served as a chemoattractant, was placed in the lower chamber. All experiments were performed in triplicate. The Matrigel angiogenesis assay was performed as described previously [15]. The human umbilical vein endothelial cells (HUVECs) were cultured with DMEM supplemented with 10% fetal bovine serum with or without 0.2 mg/ml CD151 mAb 9B.

### Cell proliferation assay

Cell proliferation was analyzed using a 3-(4,5)- dimethylthiahiazo (-z-y1)-3,5-di- phenytetrazoliumromide (MTT) assay as previously described [14]. These cells were raised with DMEM supplemented with 10% fetal bovine serum and 0.2 mg/ml CD151 mAb 9B or IgG.

### *In vivo* metastasis assays and immunohistochemical analysis

A total of 6.0×10^6^ HCCLM3 cells were used for subcutaneous xenografts in a spontaneous metastasis assay as previously described [15]. When the tumors reached a mean tumor volume of 100 mm^3^, the mice were randomly allocated into two groups (n=6); 25 mg/kg of either CD151 mAb 9B or phosphate buffer saline (PBS) was administered intraperitoneally three times per week for two weeks, and the diameter of the xenografts was monitored twice a week. The xenografts and visceral organs, including the lungs, were examined histopathologically. Tumor volume, weight, and the total number of lung metastases were assayed as previously described [30, 31].

Mouse anti-human CD34 antibodies (1:100; DakoCytomation, Denmark) and CD151 mAb 9B were used to measure microvessel density (MVD) and CD151 expression. MVD and CD151 were evaluated as described elsewhere [15].

### Statistical analysis

The statistical analysis was performed with SPSS 16.0 (SPSS, Chicago, IL). Values are expressed as the mean ± standard deviation. Quantitative data between groups were compared using Student's t test. Categorical data were analyzed using the χ^2^ test or Fisher's exact test. *P*<0.05 was considered statistically significant.

## Abbreviations

HCC: hepatocellular carcinoma
TEM: tetraspanin-enriched microdomains
TNM: tumor node metastasis
mAb: monoclonal antibody
EMT: epithelial-mesenchymal transition
